# Improving Neuromuscular Monitoring and Reducing Residual Neuromuscular Blockade With E-Learning: Protocol for the Multicenter Interrupted Time Series INVERT Study

**DOI:** 10.2196/resprot.7527

**Published:** 2017-10-06

**Authors:** Jakob Louis Demant Thomsen, Ole Mathiesen, Daniel Hägi-Pedersen, Lene Theil Skovgaard, Doris Østergaard, Jens Engbaek, Mona Ring Gätke

**Affiliations:** ^1^ Department of Anesthesiology Herlev Hospital University of Copenhagen Copenhagen Denmark; ^2^ Department of Anesthesiology Zealand University Hospital Koege Denmark; ^3^ Department of Anesthesiology Naestved Hospital Næstved Denmark; ^4^ Department of Biostatistics University of Copenhagen Copenhagen Denmark; ^5^ Copenhagen Academy for Medical Education and Simulation Herlev Hospital University of Copenhagen Copenhagen Denmark

**Keywords:** e-learning, neuromuscular monitoring, objective neuromuscular monitoring, quantitative neuromuscular monitoring, residual neuromuscular blockade, protocol

## Abstract

**Background:**

Muscle relaxants facilitate endotracheal intubation under general anesthesia and improve surgical conditions. Residual neuromuscular blockade occurs when the patient is still partially paralyzed when awakened after surgery. The condition is associated with subjective discomfort and an increased risk of respiratory complications. Use of an objective neuromuscular monitoring device may prevent residual block. Despite this, many anesthetists refrain from using the device. Efforts to increase the use of objective monitoring are time consuming and require the presence of expert personnel. A neuromuscular monitoring e-learning module might support consistent use of neuromuscular monitoring devices.

**Objective:**

The aim of the study is to assess the effect of a neuromuscular monitoring e-learning module on anesthesia staff’s use of objective neuromuscular monitoring and the incidence of residual neuromuscular blockade in surgical patients at 6 Danish teaching hospitals.

**Methods:**

In this interrupted time series study, we are collecting data repeatedly, in consecutive 3-week periods, before and after the intervention, and we will analyze the effect using segmented regression analysis. Anesthesia departments in the Zealand Region of Denmark are included, and data from all patients receiving a muscle relaxant are collected from the anesthesia information management system MetaVision. We will assess the effect of the module on all levels of potential effect: staff’s knowledge and skills, patient care practice, and patient outcomes. The primary outcome is use of neuromuscular monitoring in patients according to the type of muscle relaxant received. Secondary outcomes include last recorded train-of-four value, administration of reversal agents, and time to discharge from the postanesthesia care unit as well as a multiple-choice test to assess knowledge. The e-learning module was developed based on a needs assessment process, including focus group interviews, surveys, and expert opinions.

**Results:**

The e-learning module was implemented in 6 anesthesia departments on 21 November 2016. Currently, we are collecting postintervention data. The final dataset will include data from more than 10,000 anesthesia procedures. We expect to publish the results in late 2017 or early 2018.

**Conclusions:**

With a dataset consisting of thousands of general anesthesia procedures, the INVERT study will assess whether an e-learning module can increase anesthetists’ use of neuromuscular monitoring.

**Trial Registration:**

Clinicaltrials.gov NCT02925143; https://clinicaltrials.gov/ct2/show/NCT02925143 (Archived by WebCite® at http://www.webcitation.org/6s50iTV2x)

## Introduction

Muscle relaxants facilitate endotracheal intubation under general anesthesia and improve surgical conditions. Postoperative residual neuromuscular blockade, or residual blockade for short, occurs if the effects of the relaxant have not subsided or have not been sufficiently reversed before the patient is awakened [1]. Volunteer studies have shown partial paralysis to be associated with general muscle fatigue, double vision, and speech difficulty [2,3]. In clinical studies, residual blockade increased the risk of respiratory complications such as airway obstruction, hypoxemia, and tracheal reintubation as well as the risk of a prolonged length of stay in the postanesthesia care unit [4-7].

The depth of the neuromuscular blockade can be monitored intraoperatively with a neuromuscular monitoring device that measures the muscle response to peripheral nerve stimulation. Typically, the response of the adductor pollicis muscle, adducting the thumb, is measured using an acceleromyographic neuromuscular monitor following stimulation of the ulnar nerve at the level of the wrist [8]. In clinical studies, this type of objective neuromuscular monitoring reduced the incidence of residual blockade and symptoms of muscle weakness [9-11]. Hence, for more than a decade, experts in the field have recommended routine neuromuscular monitoring when administering muscle relaxants [12-15]. Nevertheless, surveys reveal that many anesthetists do not routinely apply neuromuscular monitoring, even if the equipment is available [16-18]. In particular, clinicians often refrain from applying neuromuscular monitoring when administering only the short-acting muscle relaxant succinylcholine because the paralytic effect subsides within 10 minutes in most, but not all, patients. However, it was recently confirmed that even patients receiving only succinylcholine are at risk of experiencing paralysis when awakened from anesthesia, especially if not monitored with a nerve stimulator [19,20].

Repeated local educational efforts in anesthesia departments may increase the use of neuromuscular monitoring and reduce the incidence of residual blockade [21,22]. As these efforts are time consuming and require the presence of expert personnel, it is relevant to consider if an e-learning module may provide an alternative method to increase the use of neuromuscular monitoring. E-learning can achieve results similar to traditional instructional methods, but few studies have assessed the impact of e-learning on patient outcomes, possibly because of the large sample size required [23].

The aim of the INVERT study is to assess the impact of an e-learning module on neuromuscular monitoring on the application frequency of objective neuromuscular monitoring and the incidence of residual neuromuscular blockade in patients monitored with a nerve stimulator.

We hypothesize that the e-learning module will increase the use of objective neuromuscular monitoring significantly in both patients receiving succinylcholine and patients receiving a nondepolarizing muscle relaxant.

## Methods

### Design

**Figure 1:**
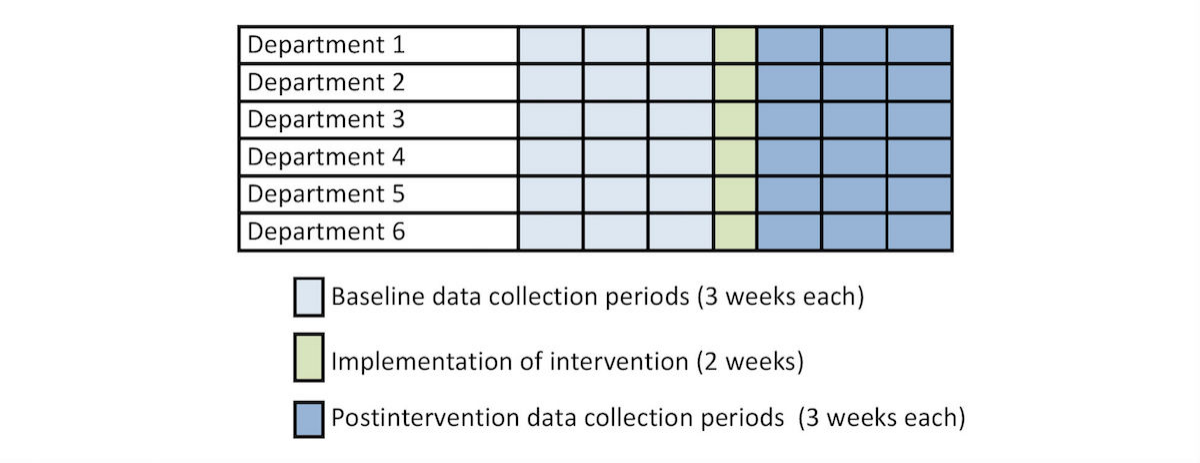
Example of data collection for an interrupted time series study.

The study is designed to assess the effect of an e-learning module on all three of the proposed levels of medical education translational research: knowledge and skills, patient care practice, and patient outcomes [24]. A “dilution” of the effect should be expected in the process of changing clinicians’ knowledge, in their behavior as well as actual patient outcomes [23]. Therefore, data from a large number of anesthesia procedures are likely required to obtain adequate strength. To make this feasible, we have chosen to conduct the study at all major hospitals in the Zealand Region of Denmark, where data on use of neuromuscular monitoring are automatically recorded in the anesthesia information management system MetaVision (iMDsoft^®^, Düsseldorf, Germany). Furthermore, we have chosen the interrupted time series design, in which data are collected repeatedly, at fixed intervals, before and after the intervention (Figure 1).

The effect of the intervention is analyzed statistically using segmented regression analysis, testing for changes in both the level and the trend of the outcome. The design is proposed to be the strongest quasi-experimental approach for assessing the longitudinal effect of interventions and also allows description of the timing of the effect of the intervention [25]. This design is well suited for our setting, where the number of participating departments is limited to the 6 departments of anesthesia at the teaching hospitals in the Zealand Region of Denmark, making experimental designs such as cluster randomized trials unfeasible [26]. Baseline data are being obtained from the descriptive study *“Use of neuromuscular blocking agents and neuromuscular monitoring in 7 Danish teaching hospitals—a cross-sectional study”* (NCT02914119), in which data are collected from the same hospitals in the year leading up to the intervention. The e-learning module was implemented over a period of 2 weeks, and we area again collecting data from all departments at fixed 3-week intervals.

### Eligibility

#### Departments

We are including the 6 anesthesia departments at the teaching hospitals in the Zealand Region of Denmark following agreement of the head of each department to participate.

#### Anesthesia Personnel

We are including the following anesthesia personnel: nurse anesthetists in training, certified registered nurse anesthetists, first- though fourth-year residents in anesthesiology, and certified anesthesiologists. We are excluding personnel without clinical functions, such as administrative personnel.

#### Patients

We are including and collecting data from all patients undergoing general anesthesia with neuromuscular blockade in each data collection period. Patients undergoing general anesthesia on more than one occasion will be included in the analyses as one case for every general anesthetic received.

### Intervention: E-Learning Module

**Figure 2:**
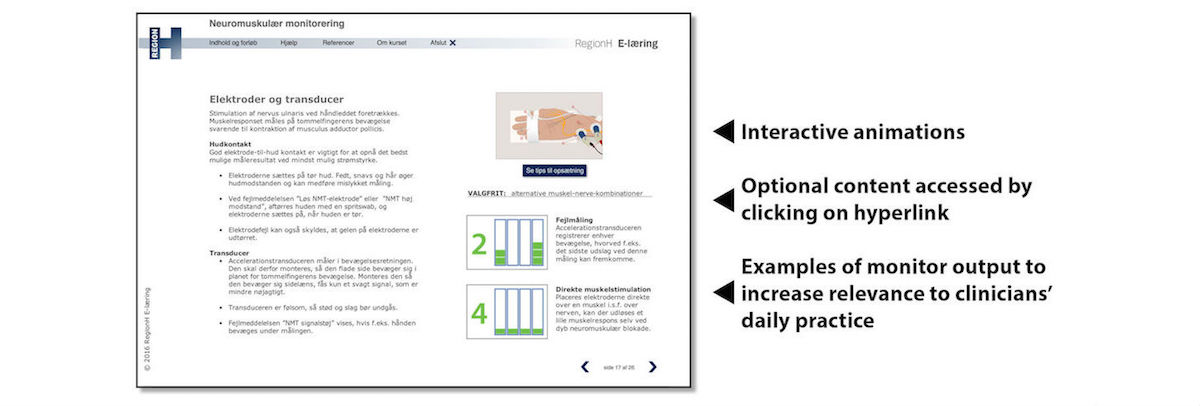
Screen shot from e-learning module.

The investigator group has developed the e-learning module in collaboration with the Regional Unit for Development and Evaluation of Learning Technologies in the Capital Region of Denmark. Learning objectives for the module are based on a needs assessment, including focus group interviews with nurse anesthetists and anesthesiologists from 5 different Danish hospitals and clinical observations of neuromuscular monitoring use in practice (NCT02239965) as well as a previous Danish survey [18] and expert opinions. The main topics in the course are as follows: (1) background and clinical consequences of residual blockade, (2) neuromuscular monitoring and stimulation patterns, (3) practical tips on monitoring and troubleshooting equipment malfunction, and (4) reversal of neuromuscular blockade. The course participants are of different educational backgrounds and have anesthesia experience ranging from short periods to decades of employment. We have aimed to target this diversity in participant background by making the e-learning module adaptable to learners’ needs, specifically by making part of the material optional (Figure 2) [27]. Furthermore, clickable animations are included to stimulate interaction and increase learning [28], while examples of monitor output make the module relevant to participants’ daily practice. The course duration is approximately 30 minutes.

### Implementation of the Intervention

We recruited a local investigator at each study site. This person is responsible for motivating the anesthesia personnel to complete the e-learning module and introduce all new employees to it. On the first day of implementation, the local investigators introduced the e-learning module. The primary investigator also visited the departments in the intervention period and introduced the study, but without revealing the primary outcome to the participants. The participating departments ensured that the personnel could complete the module during their normal work hours, such as by having colleagues cover for them while they complete the module. Course progress was logged for each anesthetist, and email reminders were sent to ensure the highest possible completion rate. The local investigator and the head of department were also informed about the completion rate throughout the implementation period. To increase awareness about the study and the e-learning module, the local investigators distributed candies and pens with the INVERT study logo at intervals. New employees joining the departments after the intervention period were also included in the study. The module is available for ongoing access, meaning that learners can still access the module, such as for use as a clinical troubleshooting tool. All anesthetic practices and medication use are at the discretion of the individual anesthetist according to local guidelines.

### Blinding

Given the nature of the intervention, it was not possible to blind the anesthesia personnel. However, they are not informed of the specific outcomes, only that the study “aims to assess the effect of the e-learning module on use of neuromuscular monitoring”. The investigators in charge of data analysis will not have access to the data until after the final data collection period.

### Outcomes

We will assess the effect of the e-learning module on knowledge, behavior, and patient outcomes. A meta-analysis of studies on the effect of Web-based learning indicates that an e-learning module will have greatest effect on knowledge, a smaller effect on skills, and a yet smaller effect on behavior and patient-related outcomes [23]. We have chosen the primary outcome based on what we believe to be of highest clinical relevance, balanced with the feasibility of collecting data on this outcome from thousands of patients.

### Primary Outcome

- Application of neuromuscular monitoring in cases involving succinylcholine only
- Application of neuromuscular monitoring in cases involving a nondepolarizing muscle relaxant (with or without succinylcholine)

The primary outcome is divided because the two types of relaxants are used in different clinical situations: succinylcholine for “rapid sequence induction and intubation” and nondepolarizing relaxants for nonemergent tracheal intubation.

### Secondary Outcomes

- Last recorded train-of-four (TOF) ratio (a stimulation pattern commonly used in neuromuscular monitoring, where a ratio < 0.9 is defined as residual blockade) before tracheal extubation in patients receiving a nondepolarizing relaxant
- Administration of the reversal agent sugammadex or neostigmine and repeated administration of the reversal agent
- Timing and dosage of reversal with sugammadex or neostigmine in relation to the neuromuscular monitoring values
- Time from administration of the reversal agent (sugammadex or neostigmine) to extubation
- Time from tracheal extubation or removal of the supraglottic airway device to discharge from the postanesthesia care unit in cases involving a nondepolarizing relaxant with or without neuromuscular monitoring
- Pre- and postcourse test scores as a measure of course participants’ knowledge

Secondary analyses that may be performed if the data allow it include the incidence of respiratory complications, arterial oxygen desaturation, and the effect of the e-learning module at the individual level.

### Data Collection

Data are recorded in the MetaVision anesthesia information management system either automatically or manually, by the anesthetist. TOF data are recorded automatically when the neuromuscular monitoring module is activated. The anesthesia information management system saves TOF data every minute, even if the neuromuscular monitoring module measures a TOF value every 12 seconds. To ensure that the reported TOF values at the time of tracheal extubation are not erroneously measured due to lack of fixation of the patient’s hand, movements caused by the surgical personnel, or other factors, we will collect the last five TOF values before tracheal extubation and use the highest value, given that the values increase incrementally. We will develop an algorithm for choosing the last correctly measured TOF value and will perform manual validation on part of the dataset. Data manually entered into the anesthesia information management system include the type and dose of intravenous drugs and the time of tracheal extubation. The anesthetist enters these data by pressing a button in the software and can alter the time and the details, such as the dose of medicine administered, afterward if needed. Where possible, we will seek to validate these manually entered data, such as by comparing them to changes in ventilatory data from the anesthesia machine. In case of doubt about the correctness of data for a particular case, we will access the full dataset for that case in order to investigate further.

### Sample Size Calculation and Statistical Analysis

A simulation-based sample size calculation revealed that the sample size necessary to detect an increase in neuromuscular monitoring from 40%-60% (with an 80% power) would require 3 consecutive data collection periods, each comprising approximately 100 patients per site, both before and after the implementation of the intervention. We expected to achieve this sample size by letting each period be 3 weeks long. The baseline value of 40% was based on a 1-month data sample from quality assurance at the participating hospitals, while the increase to 60% reflects the minimal effect size that we believe would make it worthwhile to disseminate the course to other departments. It is possible that the effect of the module could “wear off” after the 9 weeks of postintervention data collection if either a loss of knowledge occurs or the motivation to use neuromuscular monitoring decreases after the study period. To assess this, we extended the postintervention data collection period beyond what the sample size calculation indicated. This extension is ethically justifiable because prolonging the data collection does not affect patient treatment and because the data are already automatically collected in the anesthesia information management system.

When analyzing the data, we will use segmented regression analysis [25]. All results will be reported with 95% confidence intervals when relevant. We will test continuous variables for normality by visual inspection before summarization with the mean (standard deviation). Non-normally distributed continuous variables and ordinal variables will be described as the median (95th percentile range) and categorical variables as the number (percentage), if not otherwise specified. Analyses will be performed using SPSS version 22 (SPSS Inc., Chicago, IL, USA). The investigators JLT and LTS will conduct the analyses. We will consider a two-tailed *P* value of less than 0.05 to be statistically significant.

### Missing Data

In the analysis of the secondary outcomes, we will exclude cases with missing data on the outcome studied, that is, we will perform only per-protocol analyses. However, in reporting of the median TOF ratio at tracheal extubation, we will include both an analysis where a TOF count between 0 and 3 (and hence no TOF ratio reported) is included as a TOF ratio of 0 and a per-protocol analysis where only cases with a TOF ratio are included. Furthermore, we will report the proportion of patients transferred from the operating room while still tracheally intubated and, if possible, report the cause for this, such as severe residual neuromuscular blockade.

### Ethical Considerations and Study Registration

The study solely uses data routinely registered in the anesthesia information management system. The Committees on Health Research Ethics for the Capital Region of Denmark have confirmed that the study does not need approval from the committee system and that there is no need for individual patient consent (protocol no. 16028220). The study is registered at clinicaltrials.gov (NCT02925143) and with The Danish Data Protection Agency (HGH-2015-063/04364).

## Results

All 6 anesthesia departments agreed to participate in the study. The e-learning module was implemented simultaneously in all departments on 21 November 2016. Currently, postintervention data are being collected. We expect to publish the results in late 2017 or early 2018.

## Discussion

We have designed a study that aims to assess the value of an e-learning module on neuromuscular monitoring at all levels of potential effect: knowledge and skills, patient care practice, and patient outcomes. We expect that the multicenter design and the large number of participants completing the e-learning module will increase the internal and external validity of results. The primary outcomes are automatically recorded in the anesthesia information management system, increasing the validity of the collected data.

While randomized controlled trials are the gold standard of design in clinical research, this design is sometimes unfeasible in medical education research. Participants, in our case anesthesia personnel, completing the e-learning module cannot be randomized individually to use or not use the knowledge that they gained from the module. Similarly, while it is practically possible to randomize patients at a particular hospital to receive treatment from either an anesthetist who had completed the module or one who had not, there would be a nesting effect that could affect the results, such as due to local traditions regarding neuromuscular monitoring. The effect of the intervention could also spill over from anesthetists in the intervention group to the control group, such as through teamwork or meetings. One way of overcoming this challenge would be to conduct a cluster randomized trial [26], but, as mentioned earlier, this was not possible because of the limited number of potential participating departments. It is important to note, however, that the INVERT study aims to answer the question “can use of neuromuscular monitoring be increased by implementing an e-learning module on the subject?”, as opposed to assessing the effect of e-learning in general. Therefore, the interrupted time series design may very well be the optimum choice of design [25]. Furthermore, the baseline study describing use of neuromuscular monitoring in the participating departments in the year leading up to implementation of the e-learning module may, to some degree, account for not having a control group by describing an underlying trend in use of monitoring (ie, the departments function as their own historical controls).

There will be some limitations to the study. The anesthetist manually enters certain data in the anesthesia information management system, which should be taken into account when interpreting the results. Studies reporting on the incidence of residual neuromuscular blockade typically measure the TOF ratio upon arrival to the postanesthesia care unit. As this approach would require the presence of investigators in all departments at all times throughout the study period, it was not possible in our study. Instead, we chose to use the data that were readily available from the anesthesia information system, namely, the last recorded TOF ratio in the operating room. For this reason, we will not obtain data on this secondary outcome for patients without monitoring with a nerve stimulator. Finally, we did not design the study to assess the effect of the e-learning module at the individual participant level, but rather at the levels of each department, as a whole. However, if possible with the data obtained, the former will described in a secondary analysis.

### Perspectives

One of the advantages of Web-based learning, such as an e-learning module, is that participant numbers can be increased with only small expenses [29]. If the e-learning module proves effective in increasing the use of neuromuscular monitoring, it may be implemented in all Danish anesthesia departments or in the training of anesthesiologists and nurse anesthetists both nationally and internationally.

In conclusion, with a dataset consisting of thousands of general anesthesia procedures, the INVERT study will thoroughly assess whether an e-learning module can increase anesthetists’ use of neuromuscular monitoring.

## Abbreviations

TOF: train-of-four
